# Association of Dietary Patterns with Incident Type 2 Diabetes Among Migrant and Nonmigrant Ghanaian Populations in the Prospective Research on Obesity and Diabetes in African Migrants (RODAM-Pros) Study

**DOI:** 10.1016/j.cdnut.2026.107652

**Published:** 2026-01-31

**Authors:** Lambert Tetteh Appiah, Mary Nicolaou, Eva L van der Linden, Felix P Chilunga, Erik Beune, Karlijn AC Meeks, Samuel N Darko, Ellis Owusu-Dabo, Bert-Jan van den Born, Charles Agyemang

**Affiliations:** 1 Department of Public and Occupational Health, Amsterdam University Medical Center (UMC), University of Amsterdam, Amsterdam, The Netherlands; 2 Department of Medicine, School of Medicine and Dentistry, Kwame Nkrumah University of Science and Technology (KNUST), Kumasi, Ghana; 3 Department of Vascular Medicine, Amsterdam Cardiovascular Sciences, Amsterdam UMC, University of Amsterdam, Amsterdam, The Netherlands; 4 Division of Endocrinology, Diabetes, and Nutrition, Department of Medicine, University of Maryland School of Medicine, Baltimore, MD, United States; 5 Department of Epidemiology and Public Health, University of Maryland School of Medicine, Baltimore, MD, United States; 6 School of Public Health, KNUST, Kumasi, Ghana; 7 Department of Internal Medicine, Section Nephrology, Amsterdam Cardiovascular Sciences, Amsterdam UMC, University of Amsterdam, Amsterdam, The Netherlands

**Keywords:** dietary patterns, type 2 diabetes mellitus, migrants and nonmigrants, Ghanaian, RODAM-Pros study

## Abstract

**Background:**

Limited longitudinal knowledge exists between dietary patterns (DPs) in Africans and the incidence of type 2 diabetes (T2D).

**Objective:**

We investigated the association between 3 prevalent DPs among Ghanaians and incident T2D across geographical contexts

**Methods:**

One thousand three hundred and fifty-three participants from the prospective Research on Obesity and Diabetes in African Migrants study were followed up for a median duration of 6.7 (6.4, 6.9) y. Three previously established DPs from our baseline data (i.e., “mixed pattern,” “animal pattern,” and “roots and tubers pattern”) were used, and T2D incidence was determined based on World Health Organization criteria. Poisson regression models were used to analyze the associations between terciles of these DPs and incident T2D at follow-up among the entire population, by geographical context, and by sex, accounting for important covariates.

**Results:**

The study revealed a significant association between the “animal DP” and incident T2D among the total population compared to the lower tercile of intake [incidence rate ratio (IRR) was 2.72; 95% confidence interval (CI): 1.18, 6.28 in the upper tercile and 3.33; 95% CI: 1.55, 7.17 in the middle tercile]. However, when disaggregated by study site (rural Ghana, urban Ghana, and Amsterdam), these associations did not reach statistical significance, likely due to reduced statistical power in the stratified analyses. There was significant interaction between animal DP and sex, with increased risks among females (IRR: 6.76; 95% CI: 1.36, 39.5). Also, a higher intake of root, tubers, and plantain DP tercile was associated with a lower risk of T2D, although not statistically significant.

**Conclusions:**

We observed a positive association between adherence to an animal DP and the incidence of T2D, particularly among females. Dietary modifications toward a lesser consumption of an animal-pattern diet may reduce risk of T2D at the population level.

## Introduction

Risk of type 2 diabetes mellitus (T2D) is on the rise in Africa, with an estimated 24 million individuals living with diabetes in Africa according to the International Diabetes Federation 2022 report [1,2]. Indeed, the WHO projects that ∼55 million Africans are at risk of getting diabetes by 2045, translating into an alarming 134% increase compared with the 2021 data [3]. Sadly, ≤46% of individuals with diabetes in Africa know their status, although this may vary widely across the continent, predisposing them to risk of severe illness and associated 3‒4-fold higher all-cause and Cardiovascular Disease (CVD) mortality compared to nondiabetes mortality worldwide [4,5].

Published data support an increasing burden of T2D among African migrants in Europe [6], with accumulating evidence indicating that sub-Saharan African (SSA) migrants, particularly West Africans, are disproportionally affected. In a recent meta-analysis, the prevalence of T2D was nearly 3 times higher in SSA migrants than in European host populations [6,7], whereas findings from the Research on Obesity and Diabetes in African Migrants (RODAM) show that T2D affects 5% of adult Ghanaians in rural areas, 10% in urban areas, and 8%‒15% of migrant populations in Europe [6]. The high prevalence of T2D in both SSA and their migrant counterparts is due in part to the rising prevalence of obesity driven mainly by the adoption of a sedentary lifestyle, and the shift in diet toward poor eating habits occasioned by factors such as migration and urbanization [8,9].

Dietary patterns (DPs) are an important modifiable lifestyle factor associated with diabetes, because they are linked to increased overweight and obesity, which heighten the development of T2D [10]. DPs characterized by high intakes of chocolate confectionery, butter, low-fiber, added sugars, and low fresh fruits/vegetables are associated with a higher incidence of T2D [11], whereas DPs characterized by rice, meat, fruits, and vegetables have been linked to a reduced risk of T2D [12,13]. DPs and the incidence of T2D are also known to differ according to geographical setting, and a number of studies have contributed to this understanding [12,[14], [15], [16]]. International migration and urbanization in SSA might cause food preferences to shift from a more traditional diet to a more westernized one with its attendant possible diabetogenic risk [8,17,18]. Additionally, an individual’s DPs may largely remain unchanged in adulthood and may influence the risk of developing certain health conditions, such as T2D, later in life [19].

The association between DPs and T2D has been previously described in various cross-sectional studies [9,13], however, the cross-sectional nature of the data does not allow for causal inference. Prospective data on the association between diet and incident T2D in African populations are still lacking.

Ghana is experiencing a strong rural to urban transition, and many Ghanaians have migrated to Europe in the past 3 decades, generating an important opportunity to study the effect of migration on T2D risk [20,21]. This study, therefore, sought to examine temporality in associations between baseline DPs and incident diabetes among Ghanaians living in different geographical contexts and to explore any associated sex-specific patterns. We hypothesized that there is an association between adherence to specific DPs and the incidence of T2D, which may vary by geographical context and by sex.

## Methods

### Study population and design

This study utilized data from the prospective RODAM study (RODAM-Pros), a prospective cohort study nested within the baseline RODAM study (2012‒2015). The overarching goal was to identify key changes in environmental exposures and epigenetic modifications responsible for the high burden of cardiovascular disease risk among African migrants in the diaspora that share a common geographic origin [22]. Details of the methods and design have been provided elsewhere [22]. In brief, the RODAM-Pros study is based on the baseline participants of the RODAM study (2012‒2015), whose data were collected at follow-up (2019‒2021), and is restricted to the Netherlands, rural and urban Ghana, because the recruitment strategies in these sites allowed participants to be followed over time.

All participants who completed the baseline RODAM assessment (adult residents aged ≥25 y) were eligible for this current study [23]. Excluded participants included those who were already diagnosed with diabetes and had no data on dietary intake. In the follow-up, study participants were contacted by phone and through home visits for the follow-up data collection [24]. If there were no means of contact available for a participant (e.g., because of changed phone numbers), then the team relied on the household head’s contact details or the community members in each specific enumeration area to reach the participant. If a potential participant had migrated internally (to another village or city), the participant’s contact details were used as a means to reach them.

In Amsterdam, the Netherlands, all Ghanaian participants who participated in the baseline RODAM study and consented to participate in future research were invited for follow-up examination. Efforts were made to increase the response rate through repeated phone calls by the research team if individuals did not respond to the initial written invitation. In Ghana, the research team visited several houses in both rural and urban Ghana to motivate participants to take part in the study. Furthermore, community sensitizations were carried out through radio, television, and community organizations such as churches and African mosques to create awareness about the importance of participating in the follow-up.

### Ethical consideration

Ethical approvals were obtained from respective ethics committees in Ghana [School of Medical Sciences/Kwame Nkrumah University of Science and Technology, Publication and Ethics Review Board (reference CHRPE/AP/172/19)] and the Netherlands (Institutional Review Board of the Amsterdam Medical Center (AMC), University of Amsterdam, reference NL32251.018.10).

### Measurements

#### Nutritional assessment and DP

In brief, we used a Ghana-food propensity questionnaire (Ghana-FPQ) to query the usual intake frequencies of 134 food items in predefined portion sizes over the past 12 mo. Energy intake was calculated using the West African Food Composition Table and the German Nutrient Database (Bundeslebensmittelschlusse (BLS) 3.01, 2010) [13], which translated the usual food intake into total energy (kilocalories per day). The 134 food items in the Ghana-FPQ were combined into 30 food groups following their nutrient profile and culinary use.

We utilized previously identified DPs derived from the baseline RODAM study as exposure variables [23]. Details of the DP analysis have been published elsewhere [13]. Following that, DPs were identified by employing principal component analysis using the powerful procedure for exploratory factor anaylsis and ddata reduction (PROC FACTOR) procedure [13]. This method identified principal components that explain the maximum of the total variance of food intake. The factors were orthogonally rotated (varimax rotation) to ensure that they remained uncorrelated, facilitating their interpretability [13]. Three DPs were identified: a “mixed” DP (explained 14.4% of the total variance), a “roots, tubers and plantain” DP (explained 5.7% of the total variance), and an “animal products” DP (explained 8.8% of the total variance). The “mixed pattern” was defined by low intake of vegetarian mixed dishes and palm oil and high intakes of whole-grain cereals, sweet spreads, dairy products, potatoes, vegetables, chicken, coffee and tea, soda and juices, and condiments. The “animal pattern” was characterized by high intakes of dairy products, red meat, processed meat, eggs, legumes, rice and pasta, fish, meaty mixed dishes, cakes, sweets and condiments, whereas the “roots and tubers pattern” was characterized by high intake of refined grains, fruits, nuts, seeds, roots, tubers, and plantains, fermented maize products (kenkey and banku), legumes, and palm oil. Each subject was allocated a score for the identified DPs to rate the subjects according to DP adherence; higher scores were indicative of higher adherence.

#### Assessment of T2DM

At both baseline and follow-up, fasting blood samples were collected following standard procedures by trained research assistants during the time of physical assessments. Samples were processed and divided into aliquots right after collection and temporarily stored at −20°C, before transportation to the local research centers, where the samples were kept at −80°C. Later, the samples were shipped to the analytical laboratory (Amsterdam, The Netherlands) for measurement of biochemical parameters. Fasting plasma glucose was measured in venous blood (fluoride; ABX Pentra 400 chemistry analyzer; HORIBA ABX SAS). T2DM was defined following the WHO diagnostic criteria (fasting plasma glucose ≥7.0 mmol/L, current use of medication prescribed to treat diabetes, or self-reported diabetes) [22] or a glycated hemoglobin >6.5% [25]. Incident T2DM was defined as patients who were free of T2DM at baseline per the above stated criteria who developed T2DM at follow-up.

#### Assessment of covariates

Each participant completed a general questionnaire on socio-demographics and lifestyle. This was done face-to-face by trained research personnel or self-administered based on the participant’s preference. Educational level assessment was based on local circumstances and subsequently harmonized to encompass 4 categories: never been formally educated; lower vocational schooling or secondary schooling; intermediate vocational schooling or intermediate/higher secondary schooling; and higher vocational schooling or university. The WHO STEPwise approach to chronic disease risk surveillance (STEPS) questionnaire [26] was adopted to measure physical activity (metabolic equivalent time in hours per week), which included physical activity at work, while commuting, and in leisure time. Smoking was categorized as current, former, or nonsmoker. Height (centimeters) was measured using a portable stadiometer, weight (kilogram) with a digital scale, and waist circumference (centimeters) with a measuring tape (all devices SECA), in light clothes without shoes. BMI was computed as weight divided by height squared (in kg/m^2^) [22]. Following a minimum of 5 min of rest, blood pressure (BP) was recorded 3 times on the participant’s arm while seated, utilizing a correctly sized cuff and a semi-automated oscillometric equipment (Microlife WatchBP Home; Microlife AG). The average of the second and third measurements was utilized in the analysis. Hypertension was defined as systolic BP (SBP) ≥140 mmHg, and/or diastolic BP ≥90 mmHg, and/or the use of BP-lowering medication [27].

### Data management and analysis

For the final RODAM-Pros population included in the analysis, descriptive data were summarized as percentages for categorical variables, mean ± SD for normally distributed continuous variables, and median (IQR) for nonnormally distributed variables. Participants with existing T2DM at baseline were excluded from this study before analysis. Participants excluded due to existing T2D were comparable in terms of sex and key clinical markers. However, as shown in Supplementary Table 1, participants excluded due to missing dietary data (*n* = 507) differed significantly from the analytical sample; they were younger (44 compared with 46 y, *P* < 0.001), had higher SBP (133 compared with 126 mmHg, *P* < 0.001), and had a higher BMI (28.1 compared with 25.9, *P* < 0.001). Poisson regression analysis with robust SEs was conducted to assess the association between DPs and the incidence of diabetes. This approach was chosen to provide conservative SE estimates accounting for potential model misspecification or overdispersion with time to onset as an offset term to assess the association between DPs and incidence of diabetes while adjusting for potential confounders. Four regression models were computed first for all sites combined and also stratified by study sites: rural, urban Ghana, and a migrant sample in Amsterdam. Model 1 is adjusted for age and sex; model 2: model 1 plus education level (never or elementary; low; intermediate; high vocational), total energy intake (kilocalories per day), smoking (never; former; current), and physical activity (metabolic equivalent time in hours per week), and model 3: model 2 plus, waist-hip-ratio – as per the WHO thresholds of ≥0.90 cm in males and ≥0.85 cm in females, total cholesterol and SBP (millimeters of mercury). For the present analysis, we categorized participants into terciles (lower, middle, and upper) based on the distribution of each DP score in the total sample. The lowest tercile served as the reference category in all regression models. The analyses were stratified by study site and also by sex to investigate the contextual and sex differences in DPs and T2D, respectively. For all statistical tests, a 2-sided *P* value of <0.05 was considered statistically significant. The outputs were reported as incidence risk ratios (IRRs) with a corresponding 95% confidence intervals (95% CI). Data analysis was conducted using R statistical software version 4.3.1 [28].

## Results

### Recruitment of participants

**FIGURE 1:**
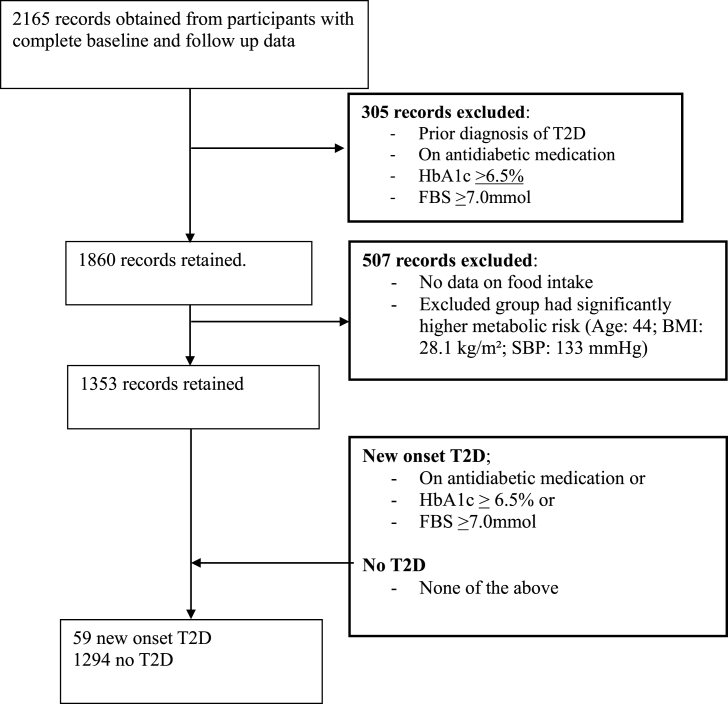
Flow chart of participant selection for the current analysis examining dietary patterns and incident type 2 diabetes (T2D) in the RODAM-Prospective study. FBS, fasting blood sugar; HbA1c, glycated hemoglobin; RODAM, Research on Obesity and Diabetes among African Migrants; SBP, systolic blood pressure.

**FIGURE 2:**
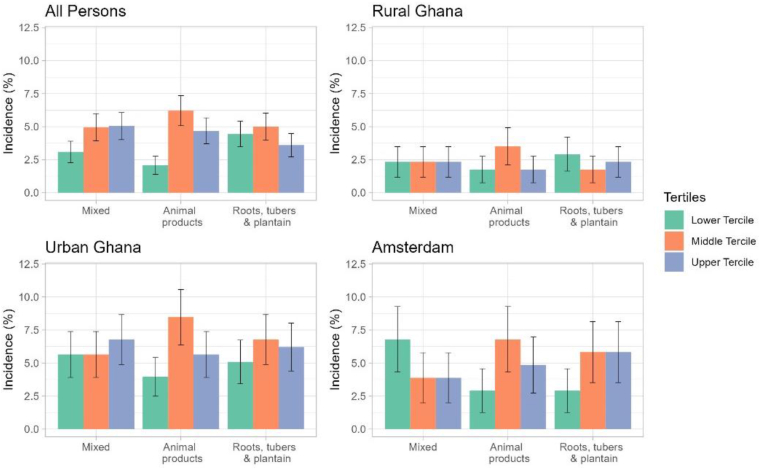
Incidence of T2D stratified by dietary patterns and by site location. T2D, type 2 diabetes mellitus.

As depicted in Figure 1, a total of 2165 participants completed the RODAM-Pros study. Out of that, 305 were excluded at baseline. From the remaining 1860, a total of 507 had no data on food intake, leaving a total of 1353 for analysis. Participants excluded were comparable in terms of sex, total cholesterol concentrations, waist-hip ratio, and the incidence of diabetes; however, participants with missing data on food intake were significantly younger (44 compared with 46 y), had higher SBP (133 mmHg compared with 126 mmHg) and a higher BMI (28.1 compared with 25.9) as shown in Supplementary Table 1. Figure 2 compares how the different DPs are associated with the IRR of T2D across various study locations. The IRR of T2D is generally lowest in rural Ghana compared to the other sites, whereas animal diet DP appears to be associated with the highest incidence of T2D across all sites.

### Descriptive statistics

Table 1 presents the key socio-demographic, lifestyle, and anthropometric characteristics of participants in the study. The median age (IQR) of the study participants was 46 (38, 54) y and the participants from urban Ghana were slightly younger than those in rural Ghana and in Amsterdam. There were more females (65.8%) than males, particularly in urban Ghana (70.4%), who participated in this study. Educational level was highest in Amsterdam Ghanaians (7.1%) and lowest in rural Ghana (1.9%). SBP and the percentage of individuals with hypertension were highest in Amsterdam compared to the remaining study sites, whereas serum total cholesterol and LDL cholesterol were highest in urban Ghana (5.13 mmol/L and 3.41 mmol/L, respectively). Overall, most participants were overweight, with the prevalence of overweight and smoking being highest in Amsterdam than in other sites. Rural Ghanaians were of normal BMI. Total energy intake was highest in rural Ghana and lowest in urban Ghana, whereas physical activity was highest in Amsterdam. The overall incidence of T2D across study sites was (4.4%). The crude incidence of T2D was higher in urban Ghana (6.0%) than in Amsterdam (4.9%) and rural Ghana (2.3%) (Table 1).

**TABLE 1:** Baseline characteristics of study cohort stratified by site

Characteristic	Overall, *n* = 1353^1^	Study site		
		Rural Ghana, *n* = 513^1^	Urban Ghana, *n* = 531^1^	Amsterdam, *n* = 309^1^
Age, y	46 (38, 54)	47 (37, 56)	45 (37, 53)	47 (41, 53)
Years of follow-up	6.7 (6.4, 6.9)	6.7 (6.5, 6.8)	6.8 (6.5,6.9)	6.4 (5.9, 6.8)
Sex				
Male	463 (34.2)	183 (35.7)	157 (29.6)	123 (39.8)
Female	890 (65.8)	330 (64.3)	374 (70.4)	186 (60.2)
Educational level completed				
Never or primary	606 (44.8)	312 (60.8)	199 (37.5)	95 (30.7)
Low	520 (38.4)	159 (31.0)	241 (45.4)	120 (38.8)
Intermediate	170 (12.6)	32 (6.2)	66 (12.4)	72 (23.3)
Higher	57 (4.2)	10 (1.9)	25 (4.7)	22 (7.1)
Systolic blood pressure (mmHg)	124 (113, 136)	119 (110, 134)	122 (113, 135)	130 (121, 140)
Hypertension	475 (35.1)	144 (28.1)	177 (33.3)	154 (49.8)
Use of antihypertensives	138 (10.2)	31 (6.0)	45 (8.5)	62 (20.1)
Serum total cholesterol, mmol/L	4.81 (4.02, 5.51)	4.26 (3.62, 5.09)	5.13 (4.48, 5.84)	4.89 (4.23, 5.56)
Serum HDL cholesterol, mmol/L	1.24 (1.04, 1.49)	1.17 (0.97, 1.40)	1.24 (1.07, 1.46)	1.42 (1.18, 1.64)
Serum LDL cholesterol, mmol/L	3.02 (2.37, 3.71)	2.63 (2.08, 3.27)	3.41 (2.73, 4.01)	3.03 (2.59, 3.69)
Smoking				
Yes	28 (2.1)	13 (2.5)	5 (0.9)	10 (3.2)
No, I have never smoked	1252 (92.5)	470 (91.6)	501 (94.4)	281 (90.9)
No, but I used to smoke	73 (5.4)	30 (5.8)	25 (4.7)	18 (5.8)
BMI, kg/m^2^	25.3 (21.7, 29.3)	22.1 (19.5, 24.7)	26.9 (23.8, 31.2)	28.1 (25.5, 30.7)
METs - h/wk	90 (25, 192)	90 (36, 168)	80 (12, 168)	125 (31, 288)
Total energy (kcal/d)	2434 (1955, 3073)	2666 (2107, 3519)	2271 (1918, 2753)	2430 (1834, 3114)
Waist-to-hip ratio	0.90 (0.86, 0.94)	0.89 (0.85, 0.93)	0.90 (0.86, 0.94)	0.91 (0.86, 0.96)
Mixed dietary pattern	‒0.59 (‒0.88, 0.08)	‒0.73 (‒0.96, ‒0.42)	‒0.74 (‒0.93, ‒0.50)	0.78 (0.46, 1.23)
Animal products pattern Roots, tubers, and plantain dietary pattern	‒0.13 (‒0.67, 0.52) ‒0.02 (‒0.47, 0.58)	‒0.36 (‒0.88, 0.28) 0.56 (0.02, 1.34)	0.27 (‒0.27, 0.85) ‒0.12 (‒0.48, 0.24)	‒0.31 (‒0.80, 0.18) ‒0.55 (‒0.85, ‒0.18)
Incidence of T2D at follow-up	59 (4.4)	12 (2.3)	32 (6.0)	15 (4.9)

Hypertension was defined as having a SBP of ≥140 or DBP of ≥90 mmHg and/or using BP-lowering medication.

Abbreviations: BP, blood pressure; DBP, diastolic blood pressure; MET, metabolic equivalent; SBP, systolic blood pressure; T2D, type 2 diabetes.

1 Values are median (IQR) or *n* (%).

### Association between DPs and T2D incidence

Table 2 shows the association between DPs and the incidence of T2D among all study sites combined. The association between the mixed DP and incidence of T2D was not statistically significant, and this was also true for root, tubers, and plantain DP. However, there was a significant association between the animal products DP and the incidence of T2D in all the models (1‒3). The fully adjusted model showed that participants in the upper tercile of the animal product DP had higher chances of having T2D than those in the lowest tercile (IRR: 2.72; 95% CI: 1.18, 6.28, *P* = 0.019) and participants in the middle tercile of the animal product DP had higher chances of having T2D than those in lowest tercile (IRR: 3.33; 95% CI: 1.55, 7.17, *P* = 0.002).

**TABLE 2:** Association between baseline dietary patterns and incidence diabetes – all sites

Dietary pattern	Model 1					Model 2			Model 3	
	*n*	IRR^1^	95% CI^1^	*P* value	IRR^1^	95% CI^1^	*P* value	IRR^1^	95% CI^1^	*P* value
Mixed	1353									
Lower tercile		Reference	—		—	—		—	—	
Middle tercile		1.61	0.83, 3.23	0.16	**1.57**	0.79, 3.09	0.20	1.63	0.82, 3.23	0.17
Upper tercile		1.71	0.89, 3.40	0.12	1.97	0.99, 3.94	0.06	1.95	0.97, 3.93	0.06
Animal products	1353									
Lower tercile		Reference	—		**—**	—		**—**	—	
Middle tercile		3.32	1.62, 7.49	<0.01	3.41	1.60, 7.28	<0.01	3.33	1.55, 7.17	<0.01
Upper tercile		2.79	1.30, 6.50	0.01	2.86	1.26, 6.46	0.01	2.72	1.18, 6.28	0.02
Roots, tubers, and plantain	1353									
Lower tercile		Reference	—		**—**	—		**—**	—	
Middle tercile		1.11	0.61, 2.03	0.74	1.01	0.55, 1.87	0.96	1.06	0.57, 1.97	0.85
Upper tercile		0.80	0.41, 1.54	0.50	0.70	0.32, 1.52	0.37	0.85	0.39, 1.88	0.70

Model 1: age, sex, model 2: model 1 plus education (never or elementary; low; intermediate; high vocational), total energy intake (kilocalories per day), smoking (never; former; current), physical activity (METs-h per week). Model 3: model 2 plus BMI (kg/m^2^), waist-hip-ratio, total cholesterol (mmol/L), SBP (mmHg).

Abbreviations: CI, confidence Interval; IRR, incidence rate ratio; MET, metabolic equivalent; SBP, systolic blood pressure.

1 IRR, CI - means reference.

Table 3 shows the association between DPs and the incidence of T2D among the study participants stratified by study sites: rural Ghana, urban Ghana, and Amsterdam. There was no statistically significant association between DPs and incidence of T2D at the various study sites (rural and urban Ghana, and Amsterdam), both in the unadjusted and adjusted models (models 1‒3).

**TABLE 3:** Association between baseline dietary patterns and incidence diabetes at follow-up

Subgroup	Dietary pattern	Model 1				Model 2			Model 3		
		*n*	IRR^1^	95% CI^1^	*P* value	IRR^1^	95% CI^1^	*P* value	IRR^1^	95% CI^1^	*P* value
Rural Ghana											
	Mixed dietary pattern	513									
	Lower tercile		Reference	—		—	—		—	—	
	Middle tercile		1.01	0.24, 4.27	1.00	1.00	0.25, 4.04	>1.00	1.07	0.26, 4.42	0.92
	Upper tercile		1.00	0.24, 4.23	1.00	1.08	0.23, 5.18	0.92	1.23	0.26, 5.85	0.80
	Animal products	513									
	Lower tercile		Reference	—		**—**	—		**—**	—	
	Middle tercile		2.02	0.52, 9.71	0.33	1.95	0.47, 8.05	0.36	2.42	0.57, 10.2	0.23
	Upper tercile		1.02	0.18, 5.85	0.98	1.03	0.18, 5.75	0.98	1.05	0.18, 5.99	0.96
	Roots, tubers, and plantain	513									
	Lower tercile		Reference	—		**—**	—		**—**	—	
	Middle tercile		0.60	0.12, 2.44	0.48	0.65	0.15, 2.84	0.57	**0.75**	0.17, 3.38	0.71
	Upper tercile		0.79	0.20, 3.01	0.73	1.11	0.18, 6.78	0.91	1.51	0.22, 10.2	0.67
Urban Ghana											
	Mixed dietary pattern	531									
	Lower tercile		Reference	—		**—**	—		**—**	—	
	Middle tercile		1.07	0.44, 2.62	0.88	0.94	0.39, 2.30	0.89	0.94	0.38, 2.30	0.89
	Upper tercile		1.24	0.54, 2.95	0.61	1.00	0.41, 2.47	>1.00	1.04	0.42, 2.58	0.93
	Animal products	531									
	Lower tercile		Reference	—		**—**	—		**—**	—	
	Middle tercile		2.40	1.01, 6.31	0.06	2.31	0.92, 5.81	0.07	2.14	0.84, 5.49	0.11
	Upper tercile		1.87	0.70, 5.24	0.22	1.57	0.54, 4.56	0.41	1.57	0.53, 4.69	0.42
	Roots, tubers, and plantain	531									
	Lower tercile		Reference	—		—	—		—	—	
	Middle tercile		1.36	0.58, 3.34	0.49	1.16	0.47, 2.85	0.75	1.11	0.44, 2.78	0.82
	Upper tercile		1.24	0.51, 3.08	0.63	0.88	0.32, 2.45	0.81	0.87	0.31, 2.44	0.80
Amsterdam											
	Mixed dietary pattern	309									
	Lower tercile		Reference	—		**—**	—		**—**	—	
	Middle tercile		0.60	0.16, 1.98	0.41	0.59	0.17, 2.06	0.40	0.57	0.14, 2.27	0.42
	Upper tercile		0.55	0.14, 1.82	0.34	0.44	0.11, 1.77	0.25	0.66	0.15, 2.82	0.57
	Animal products	309									
	Lower tercile		Reference	—		**—**	—		**—**	—	
	Middle tercile		2.51	0.70, 11.7	0.18	2.73	0.68, 10.9	0.16	2.30	0.56, 9.45	0.25
	Upper tercile		1.98	0.48, 9.83	0.36	1.81	0.39, 8.46	0.45	1.26	0.26, 6.19	0.77
	Roots, tubers, and plantain	309									
	Lower tercile		Reference	—		**—**	—		**—**	—	
	Middle tercile		1.89	0.50, 9.00	0.37	1.81	0.44, 7.48	0.41	1.80	0.40, 8.06	0.44
	Upper tercile		1.86	0.49, 8.89	0.38	1.77	0.40, 7.77	0.45	1.71	0.38, 7.57	0.48

Model 1: age, sex, model 2: model 1 plus education (never or elementary; low; intermediate; high vocational), total energy intake (kcal/d), smoking (never; former; current), physical activity (METs-h per week). Model 3: model 2 plus BMI (kg/m^2^), waist-hip-ratio, total cholesterol (mmol/L), SBP (mmHg).

Abbreviations: CI, confidence interval; IRR, incidence rate ratio; MET, metabolic equivalent; SBP, systolic blood pressure.

1 IRR, CI - means reference.

There was a trend toward significance among urban females who adhered to an animal products DP and the risk of developing T2D (IRR: 2.8; 95% CI: 1.0, 8.3, *P* = 0.05), though this observation was not statistically significant when data were stratified by sex and by site, as seen in Supplementary Table 2A‒F.

Supplementary Table 3 depicts the interaction of animal products DP and T2D with sex. Females in the middle tercile of the animal product DP had higher chances of having T2D than those in the lowest tercile (IRR: 6.76; 95% CI: 1.36, 39.5, *P* = 0.022). No significant interaction was seen with the other 2 baseline DPs when stratified by sex and location(site) (Supplemetary Table 4A, 4B and C, and 5A‒C).

## Discussion

### Summary of key findings

We examined the longitudinal association between DPs and the incidence of T2D in Ghanaians living in different geographical contexts. Our study shows that higher adherence to an animal product DP was associated with an increased risk of T2D and that this association was comparable across the different geographical sites. The study also shows that the association was stronger in females than in male participants.

### Comparison with previous studies

Consistent with the above findings, a longitudinal study conducted in the United Kingdom revealed a significant association between DPs and the incidence of diabetes mellitus [11,16]. Similarly, in a global systematic review of prospective observational studies the authors observed that adherence to the “healthy” DPs (higher consumption of vegetables, fruits, whole grains, low-fat dairy, fish, poultry and legumes) significantly reduces the risk of developing T2D, whereas “unhealthy” DPs (high loadings of red meat, fried and processed foods, French fries and sausage, foods with high glycemic index) adversely heightened risk of T2D [29]. Plausible explanations for this association include the consumption of poor diets, such as a low-fiber diet with high glycemic index, which increases one’s risk of diabetes, and the intake of high dietary fat and meat, all of which work synergistically to heighten risk of T2D in both sexes [30,31].

This present study specifically revealed a significant association between adherence to an “animal products” DP and incident T2D. Studies across China and Europe have published similar observations, particularly for people with higher intake of red meat, processed meat, and sugar-sweetened products [[32], [33], [34]]. Animal products, particularly red meat, are known to contain high concentrations of cholesterol, iron, and saturated fat. This increases the risk of obesity, which in turn causes the development of T2D [35,36]. Given that the observed association in this study was independent of obesity, other mechanisms may be involved. High intake of red and processed meats, key components of the animal DP, provides a high load of heme iron [37]. As a pro-oxidant, heme iron may increase oxidative stress and damage β-cell function [38]. Furthermore, elevated iron stores have been linked to insulin resistance and T2D risk [39,40], even in the absence of overt iron overload [41,42]. Furthermore, processed meat often contains high concentrations of nitrates or nitrites and nitrosamine compounds, which are thought to increase T2D risk [43]. Also, advanced glycation of high-fat products and meats can enhance oxidative stress and inflammatory factors, leading to insulin resistance and increasing the possibility of developing T2D [33].

The interaction analysis of this current work indicates that females who consume the animal products DP had a higher risk of developing T2D than males. This finding is in line with a large prospective case-cohort study nested in the European Prospective Investigation into Cancer and Nutrition that revealed that among females, there is a significant association between consumption of animal products (both red meat and poultry) and incidence of T2D [33]. Also, a study conducted in Northeastern China observed that the intake of total protein, animal protein, and red meat protein was positively associated with T2D in females, but not in males [44].

The finding that females who consume an animal diet rich in protein had a higher risk of developing T2D is again in consonance with the population-based SUNSET study results, which showed that the association between serum ferritin and fasting glucose, as well as T2D, was stronger in females than in males, and this was especially the case in African-origin females [45]. It is therefore plausible that a high meat intake is likely to mean a higher iron status and or storage in females than in males, and hence a higher likelihood of developing T2D from the multiple mechanisms mentioned earlier. It is also possible that males more often adhered to a mixed DP more frequently than an animal pattern, therefore making the association less pronounced than in females.

Research has shown that plant-based DPs, rich in fruits, vegetables, and whole grains, play a pivotal role in preventing various chronic diseases, including T2D [32]. In fact, the DP of high fruits and vegetables is extremely important in preventing T2D. In this study, we found that a higher intake of root, tubers, and plantain DP tercile was associated with a lower risk of T2D, although not statistically significant. A similar observation was made in the cross-sectional RODAM study previously published [13]. Indeed, evidence points to the fact that prudent DPs characterized by higher consumption of root, tubers, and fruits and vegetables are associated with a lower risk of T2D [46]. The consumption of roots, tubers, fruits, fish, vegetables, and whole grains is known to improve glycemic control due to their low energy density, low glycemic load, high fiber, and antioxidant content [47,48]. The introduction of such favorable DPs into our routine diet and nutritional counseling services may likely benefit our fight against diabetes prevention. Although we observed a significant association between animal DP and incidence of T2D, the relatively low incidence rates may reflect the generally lower burden of T2D in Africa compared with other regions such as North America and the Middle East, where incidence rates are higher [49].

### Strengths and limitations of the study

This study’s strength resides in its novelty, as to the best of our knowledge, it is the first longitudinal study to assess the association between DPs and the incidence of T2D among the Ghanaian population. It contributes new observational evidence to our understanding of the associations of baseline DPs and the risk of T2D. Due to the cumulative effects of several dietary components, analyses of DPs, which use nutrients of concern as response variables in the causal pathway, may aid in the detection of stronger relationships. Also, the study utilized a dataset from RODAM-Pros with a relatively large sample size. Despite the above-mentioned strengths, the study has some limitations that need to be mentioned. The Ghana-FPQ has not been locally validated [50], and this can lead to measurement error, which could translate into bias [51] toward the null while underestimating the true associations. The Ghana-FPQ has been successfully used in previous publications from the RODAM study [13,50], demonstrating its utility in characterizing DPs in this population, although it is still not validated. Additionally, DP categorization in this study was identified through principal component analysis. Choices relating to the consolidation of food items into food groups require subjective decisions about which foods to combine based on nutritional similarity and culinary use, and alternative grouping strategies might yield different patterns. Additionally, the number of factors to extract and the labeling of the components could affect the reproducibility of findings [52,53]. Additionally, only baseline measurements of diet were considered in this study; although many studies conduct similar analyses, the underlying assumption is that dietary habits in adulthood tend to be relatively stable over time, particularly for overall DPs [[54], [55], [56]]. Some studies have again suggested that there is minimal change in DP and quality over time regardless of the diet quality index used, indicating a single baseline measurement may be sufficient to characterize typical patterns [54,56]. The recruitment strategy relied on re-contacting baseline participants, resulting in a convenience sample of those who were reachable and willing to participate. This introduces a high risk of selection bias, as evidenced by the fact that excluded participants (due to missing data) had significantly higher BMI and SBP than the analytical sample. Consequently, our results may not be fully generalizable to the broader Ghanaian population, particularly those with higher metabolic risk profiles. Another limitation of this study is the exclusion of 507 records due to missing food data. As these excluded participants had higher BMI and SBP (Supplementary Table 1), this selection bias may have led to an underestimation of the true association between diet and T2D (bias toward the null). Furthermore, although we observed a significant association in the overall pooled analysis, the site-specific analyses yielded wide CIs and null findings. This discordance is likely attributable to limited statistical power due to the lower number of events (*n* = 59 total) when stratified by geographical site, rather than a true lack of association in specific contexts. Similarly, the wide CIs observed in the sex-interaction analysis may suggest statistical instability, and therefore, these subgroup findings should be interpreted with caution. The power analysis, imputations of missing dietary values, and their sensitivity analysis could not be performed in this study due unavailability of data.

### Clinical significance and public health implications

In this study on the association between dietary food patterns and T2D incidence in Ghanaians living in urban and rural Ghana, and in Amsterdam, we show that individuals in the middle and upper terciles of animal product consumption had 3.33- and 2.72-times higher risk of developing T2D, respectively, compared to those in the lowest tercile. Given that DPs are modifiable, the findings of this study suggest that interventions promoting reduced intake of animal products, particularly red and processed meat, may reduce diabetes incidence among the African populations. However, these findings require confirmation in larger studies and randomized trials before definitive recommendations can be made. This is especially relevant as urbanization and economic development are driving increased consumption of animal products across Africa. From a public health perspective, the findings of this study may require the need for: *1*) culturally tailored dietary guidelines for African populations that emphasize traditional plant-based foods (roots, tubers, and plantains), whereas limiting animal product intake; *2*) nutrition education programs specifically targeting females, who showed stronger associations between animal product consumption and diabetes risk; *3*) policy interventions to maintain access to traditional healthy foods even as populations urbanize and migrate; and *4*) further research to understand how migration-related dietary changes contribute to the diabetes burden in African diaspora communities.

In conclusion, we observed a statistically significant association between higher adherence to animal products DP and the incidence of T2D, particularly among females. More prospective data are required to understand the mechanisms underlying the link between animal products DP and the incidence of T2D, providing a useful avenue for dietary preventative strategies.

## Author contributions

The authors’ responsibilities were as follows – LTA, CA, B-JvdB: designed and conducted the research; CA, B-JvdB: provided essential databases for the research. LTA: analyzed data and wrote the article; LTA had primary responsibility for final content; all authors read, edited, and contributed to the manuscript revision; and all authors: read and approved the final manuscript.

## Data availability

The data supporting the findings of this study are available from the corresponding author upon reasonable request. Access requests for the data must be submitted to the corresponding author and will be evaluated by the ethics committee of the Research on Obesity and Diabetes among African migrants study.

## Funding

The author(s) disclose receipt of the following financial support for the research, authorship, and/or publication of this article: The study was funded by the European Research Council (grant number 772244). KACM is supported by the Intramural Research Program of the NIH in the Center for Research on Genomics and Global Health (CRGGH). The CRGGH is supported by the National Human Genome Research Institute, the National Institute of Diabetes and Digestive and Kidney Diseases, the Center for Information Technology, and the Office of the Director at the NIH (1ZIAHG200362).

## Conflict of interest

The authors report no conflicts of interest.
