# Supplementary material for: Association of Dietary Patterns with Incident Type 2 Diabetes Among Migrant and Nonmigrant Ghanaian Populations in the Prospective Research on Obesity and Diabetes in African Migrants (RODAM-Pros) Study
Source: Curr Dev Nutr. 2026 Jan 31;10(3):107652. doi: 10.1016/j.cdnut.2026.107652 (PMC12945621; doi:10.1016/j.cdnut.2026.107652)
Supplement: Multimedia component 1 [file mmc1.docx]

**Supplementary Table 1** comparing characteristics of participants with and without food data

|  | | **Food Data Missing** | |  |
| --- | --- | --- | --- | --- |
| **Characteristic** | **Overall**, N = 1,860^1^ | **No**, N = 1,353^1^ | **Yes**, N = 507^1^ | **p-value**^2^ |
| **Age in years** | 46(11) | 46(11) | 44(11) | <0.001 |
| **Sex** |  |  |  | 0.20 |
| Male | 654 (35.2) | 463 (34.2) | 191 (37.7) |  |
| Female | 1,206 (64.8) | 890 (65.8) | 316 (62.3) |  |
| **Educational level completed** |  |  |  | <0.001 |
| Never or Primary | 717 (41.1) | 583 (44.7) | 134 (30.5) |  |
| Low | 670 (38.4) | 502 (38.5) | 168 (38.3) |  |
| Intermediate | 271 (15.5) | 164 (12.6) | 107 (24.4) |  |
| Higher | 85 (4.9) | 55 (4.2) | 30 (6.8) |  |
| **Site** |  |  |  | <0.001 |
| Rural Ghana | 517 (27.8) | 513 (37.9) | 4 (0.8) |  |
| Urban Ghana | 538 (28.9) | 531 (39.2) | 7 (1.4) |  |
| Amsterdam | 805 (43.3) | 309 (22.8) | 496 (97.8) |  |
| **Systolic blood pressure (mmHg)** | 128(19) | 126(19) | 133(18) | <0.001 |
| **Hypertension** | 708 (38.8) | 475 (35.1) | 233 (49.3) | <0.001 |
| **Use of antihypertensives** | 219 (12.0) | 138 (10.2) | 81 (17.1) | <0.001 |
| **Serum total cholesterol, mmol/L** | 4.87(1.10) | 4.85(1.09) | 4.94(1.13) | 0.13 |
| **Serum HDL cholesterol, mmol/L** | 1.29(0.52) | 1.25(0.45) | 1.40(0.68) | <0.001 |
| **Serum LDL cholesterol, mmol/L** | 2.99(1.12) | 3.03(1.06) | 2.87(1.29) | 0.01 |
| **Smoking** |  |  |  | <0.001 |
| Yes | 51 (2.9) | 28 (2.2) | 23 (5.2) |  |
| No, I have never smoked | 1,581 (91.3) | 1,195 (92.7) | 386 (87.1) |  |
| No, but I used to smoke | 100 (5.8) | 66 (5.1) | 34 (7.7) |  |
| **BMI, kg/m2** | 26.4(5.3) | 25.9(5.4) | 28.1(4.5) | <0.001 |
| **METs - hours/week** | 118(117) | 120(116) | 83(114) | 0.02 |
| **Total Energy (kcal/day)** | 2,675(1,142) | 2,675(1,142) | NA(NA) |  |
| **DM at Round 2** | 88 (4.7) | 59 (4.4) | 29 (5.7) | 0.20 |
| **Waist-to-hip Ratio** | 0.90(0.07) | 0.90(0.07) | 0.90(0.08) | 0.30 |
| **Mixed dietary pattern** | -0.31(0.83) | -0.31(0.83) | NA(NA) |  |
| **Animal products pattern** | -0.01(0.91) | -0.01(0.91) | NA(NA) |  |
| **Roots, tubers & plantain dietary pattern** | 0.22(1.11) | 0.22(1.11) | NA(NA) |  |
| ^1^Mean(SD); n (%) | | | | |
| ^2^Welch Two Sample t-test; Pearson's Chi-squared test | | | | |

| **Supplementary Table 2A: Association between Baseline dietary patterns and Incidence diabetes by sex – Rural Ghana (female)** | | | | | | | | | | | | |  |
| --- | --- | --- | --- | --- | --- | --- | --- | --- | --- | --- | --- | --- | --- |
| **Dietary Pattern** | **Model 1** | | | | | **Model 2** | | | **Model 3** | | | | |
|  | **N** | **IRR***^1^* | **95% CI***^1^* | **p-value** | **IRR***^1^* | | **95% CI***^1^* | **p-value** | | **IRR***^1^* | **95% CI***^1^* | **p-value** | |
| **Mixed dietary pattern by site** | 330 |  |  |  |  | |  |  | |  |  |  | |
| Lower Tercile |  | — | — |  | — | | — |  | | — | — |  | |
| Middle Tercile |  | 0.25 | 0.01, 1.71 | 0.22 | 0.25 | | 0.03, 2.24 | 0.21 | | 0.31 | 0.03, 2.99 | 0.31 | |
| Upper Tercile |  | 1.02 | 0.24, 4.35 | 0.98 | 1.12 | | 0.23, 5.40 | 0.89 | | 1.53 | 0.30, 7.86 | 0.61 | |
| **Animal products by site** | 330 |  |  |  |  | |  |  | |  |  |  | |
| Lower Tercile |  | — | — |  | — | | — |  | | — | — |  | |
| Middle Tercile |  | 2.78 | 0.58, 19.7 | 0.23 | 2.61 | | 0.48, 14.1 | 0.27 | | 2.95 | 0.53, 16.4 | 0.23 | |
| Upper Tercile |  | 1.24 | 0.14, 11.0 | 0.84 | 1.09 | | 0.13, 9.34 | 0.93 | | 1.12 | 0.13, 9.98 | 0.92 | |
| **Roots, tubers & plantain by site** | 330 |  |  |  |  | |  |  | |  |  |  | |
| Lower Tercile |  | — | — |  | — | | — |  | | — | — |  | |
| Middle Tercile |  | 0.74 | 0.15, 3.38 | 0.70 | 0.71 | | 0.15, 3.40 | 0.67 | | 0.85 | 0.17, 4.24 | 0.84 | |
| Upper Tercile |  | 0.50 | 0.07, 2.56 | 0.42 | 0.56 | | 0.05, 6.02 | 0.64 | | 0.77 | 0.06, 10.6 | 0.84 | |
| *^1^* IRR = Incidence Rate Ratio, CI = Confidence Interval — means reference.  Model 1: age, sex, Model 2: Model 1 plus education (never or elementary; low; intermediate; high vocational), total energy intake (kcal per day), smoking (never; former; current), physical activity (METs-h per week). Model 3: Model 2 plus BMI (kg/m2), waist-hip-ratio, Total cholesterol (mmol/L), SBP (mmHg) | | | | | | | | | | | | |  |
|  | | | | | | | | | | | | |  |

| **Supplementary Table 2B: Association between Baseline dietary patterns and Incidence diabetes by sex – Urban Ghana (female)** | | | | | | | | | | | | | | |
| --- | --- | --- | --- | --- | --- | --- | --- | --- | --- | --- | --- | --- | --- | --- |
| **Dietary Pattern** | **Model 1** | | | **Model 2** | | | | **Model 3** | | | |  |  |  |
|  | **N** | **IRR***^1^* | **95% CI***^1^* | | **p-value** | **IRR***^1^* | **95% CI***^1^* | | **p-value** | **IRR***^1^* | **95% CI***^1^* | | **p-value** |  |
| **Mixed dietary pattern by site** | 374 |  |  | |  |  |  | |  |  |  | |  |  |
| Lower Tercile |  | — | — | |  | — | — | |  | — | — | |  |  |
| Middle Tercile |  | 1.27 | 0.49, 3.40 | | 0.62 | 1.07 | 0.41, 2.80 | | 0.90 | 1.08 | 0.41, 2.84 | | 0.88 |  |
| Upper Tercile |  | 1.46 | 0.59, 3.78 | | 0.41 | 1.11 | 0.42, 2.93 | | 0.84 | 1.15 | 0.44, 3.05 | | 0.77 |  |
| **Animal products by site** | 374 |  |  | |  |  |  | |  |  |  | |  |  |
| Lower Tercile |  | — | — | |  | — | — | |  | — | — | |  |  |
| Middle Tercile |  | 3.26 | 1.25, 10.1 | | 0.02 | 3.10 | 1.10, 8.77 | | 0.03 | 2.88 | 1.00, 8.30 | | **0.05** |  |
| Upper Tercile |  | 2.38 | 0.80, 7.86 | | 0.13 | 1.99 | 0.59, 6.65 | | 0.27 | 2.03 | 0.59, 6.97 | | 0.26 |  |
| **Roots, tubers & plantain by site** | 374 |  |  | |  |  |  | |  |  |  | |  |  |
| Lower Tercile |  | — | — | |  | — | — | |  | — | — | |  |  |
| Middle Tercile |  | 2.13 | 0.83, 6.13 | | 0.13 | 1.79 | 0.65, 4.94 | | 0.26 | 1.66 | 0.59, 4.72 | | 0.34 |  |
| Upper Tercile |  | 1.70 | 0.63, 5.00 | | 0.30 | 1.21 | 0.38, 3.83 | | 0.74 | 1.17 | 0.37, 3.70 | | 0.80 |  |
| *^1^* IRR = Incidence Rate Ratio, CI = Confidence Interval — means reference.  Model 1: age, sex, Model 2: Model 1 plus education (never or elementary; low; intermediate; high vocational), total energy intake (kcal per day), smoking (never; former; current), physical activity (METs-h per week). Model 3: Model 2 plus BMI (kg/m2), waist-hip-ratio, Total cholesterol (mmol/L), SBP (mmHg) | | | | | | | | | | | | | | |

| **Supplementary Table 2C: Association between Baseline dietary patterns and Incidence diabetes by sex – Amsterdam (female)** | | | | | | | | | | | | | |
| --- | --- | --- | --- | --- | --- | --- | --- | --- | --- | --- | --- | --- | --- |
| **Dietary Pattern** | **Model 1** | | | | **Model 2** | | | | **Model 3** | | | |  |
|  | **N** | **IRR***^1^* | **95% CI***^1^* | **p-value** | | **IRR***^1^* | **95% CI***^1^* | **p-value** | | **IRR***^1^* | **95% CI***^1^* | **p-value** | |
| **Mixed dietary pattern by site** | 186 |  |  |  | |  |  |  | |  |  |  | |
| Lower Tercile |  | — | — |  | | — | — |  | | — | — |  | |
| Middle Tercile |  | 0.67 | 0.13, 3.03 | 0.60 | | 0.64 | 0.14, 2.93 | 0.57 | | 0.62 | 0.11, 3.60 | 0.60 | |
| Upper Tercile |  | 0.56 | 0.08, 2.89 | 0.51 | | 0.53 | 0.09, 3.31 | 0.50 | | 0.77 | 0.12, 5.05 | 0.79 | |
| **Animal products by site** | 186 |  |  |  | |  |  |  | |  |  |  | |
| Lower Tercile |  | — | — |  | | — | — |  | | — | — |  | |
| Middle Tercile |  | 6.69 | 1.14, 126 | 0.08 | | 7.14 | 0.85, 60.2 | 0.07 | | 6.44 | 0.71, 58.3 | 0.10 | |
| Upper Tercile |  | 2.23 | 0.21, 48.6 | 0.52 | | 2.24 | 0.18, 28.4 | 0.53 | | 1.34 | 0.09, 21.0 | 0.84 | |
| **Roots, tubers & plantain by site** | 186 |  |  |  | |  |  |  | |  |  |  | |
| Lower Tercile |  | — | — |  | | — | — |  | | — | — |  | |
| Middle Tercile |  | 1.40 | 0.23, 10.7 | 0.72 | | 1.39 | 0.22, 8.69 | 0.73 | | 1.19 | 0.13, 10.6 | 0.87 | |
| Upper Tercile |  | 1.71 | 0.32, 12.5 | 0.54 | | 1.69 | 0.28, 10.2 | 0.57 | | 1.99 | 0.27, 14.8 | 0.50 | |
| *^1^* IRR = Incidence Rate Ratio, CI = Confidence Interval — means reference.  Model 1: age, sex, Model 2: Model 1 plus education (never or elementary; low; intermediate; high vocational), total energy intake (kcal per day), smoking (never; former; current), physical activity (METs-h per week). Model 3: Model 2 plus BMI (kg/m2), waist-hip-ratio, Total cholesterol (mmol/L), SBP (mmHg) | | | | | | | | | | | | | |

| **Supplementary Table 2D: Association between Baseline dietary patterns and Incidence diabetes by sex** - **Rural Ghana (male)** | | | | | | | | | | | | | | | | | | | | | |  |
| --- | --- | --- | --- | --- | --- | --- | --- | --- | --- | --- | --- | --- | --- | --- | --- | --- | --- | --- | --- | --- | --- | --- |
| **Dietary Pattern** | **Model 1** | | | | | **Model 2** | | | | | | | | **Model 3** | | |  |  |  |  |  |  |
|  | **N** | **IRR***^1^* | | **95% CI***^1^* | | | | **p-value** | | **IRR***^1^* | | | **95% CI***^1^* | **p-value** | | **IRR***^1^* | | | **95% CI***^1^* | **p-value** |  |  |
| **Mixed dietary pattern by site** | 183 |  | |  | | | |  | |  | | |  |  | |  | | |  |  |  |  |
| Lower Tercile |  | — | | — | | | | — | | — — | | | | — | — | | | — | | — |  | |
| Middle Tercile |  | 225 x10^6^ | | 0.00, Inf | | | | 0.10 | | 4020 x10^6^ | | | 0.00, Inf | 1.00 | | 32, 0 x10^9^ | | | 0.00, Inf | 1.00 |  |  |
| Upper Tercile |  | 0.97 | | 0.00, Inf | | | | >1.00 | | 0.18 | | | 0.00, Inf | >1.00 | | 9.03 | | | 0.00, Inf | >1.00 |  |  |
| **Animal products by site** | 183 |  | |  | | | |  | |  | | |  |  | |  | | |  |  |  |  |
| Lower Tercile |  | — | | — | | | |  | | — | | | — |  | | — | | | — |  |  |  |
| Middle Tercile |  | 0.80 | | 0.03, 20.4 | | | | 0.87 | | 0.50 | | | 0.03, 9.81 | 0.65 | | 0.35 | | | 0.01, 12.7 | 0.57 |  |  |
| Upper Tercile |  | 0.59 | | 0.02, 16.6 | | | | 0.73 | | 0.45 | | | 0.02, 9.03 | 0.60 | | 0.06 | | | 0.00, 7.22 | 0.25 |  |  |
| **Roots, tubers & plantain by site** | 183 |  | |  | | | |  | |  | | |  |  | |  | | |  |  |  |  |
| Lower Tercile |  | — | — | |  | | — | | — | |  |  |  |  |  |  |  |  |  |  |  |  |
| Middle Tercile |  | 0.00 | | 0.00, Inf | | | | 1.00 | | 0.00 | | | 0.00, Inf | 1.00 | | 0.00 | | | 0.00, Inf | 1.00 |  |  |
| Upper Tercile |  | 2.27 | | 0.20, 25.8 | | | | 0.51 | | 4.13 | | | 0.23, 75.0 | 0.34 | | 1.90 | | | 0.05, 78.4 | 0.78 |  |  |
| *^1^* IRR = Incidence Rate Ratio, CI = Confidence Interval — means reference. Model 1: age, sex, Model 2: Model 1 plus education (never or elementary; low; intermediate; high vocational), total energy intake (kcal per day), smoking (never; former; current), physical activity (METs-h per week). Model 3: Model 2 plus BMI (kg/m2), waist-hip-ratio, Total cholesterol (mmol/L), SBP (mmHg  Model 1: age, sex, Model 2: Model 1 plus education (never or elementary; low; intermediate; high vocational), total energy intake (kcal per day), smoking (never; former; current), physical activity (METs-h per week). Model 3: Model 2 plus BMI (kg/m2), waist-hip-ratio, Total cholesterol (mmol/L), SBP (mmHg) | | | | | | | | | | | | | | | | | | | | | |  |

| **Supplementary Table 2E: Association between Baseline dietary patterns and Incidence diabetes by sex** - **Urban Ghana (male)** | | | | | | | | | | | | |
| --- | --- | --- | --- | --- | --- | --- | --- | --- | --- | --- | --- | --- |
| **Dietary Pattern** | **Model 1** | | | **Model 2** | | | **Model 3** | | | |  |  |
|  | **N** | **IRR***^1^* | **95% CI***^1^* | **p-value** | **IRR***^1^* | **95% CI***^1^* | | **p-value** | **IRR***^1^* | **95% CI***^1^* | **p-value** |  |
| **Mixed dietary pattern by site** | 157 |  |  |  |  |  | |  |  |  |  |  |
| Lower Tercile |  | — | — |  | — | — | |  | — | — |  |  |
| Middle Tercile |  | 0.30 | 0.01, 3.27 | 0.33 | 0.20 | 0.01, 2.96 | | 0.24 | 0.27 | 0.02, 3.81 | 0.33 |  |
| Upper Tercile |  | 0.37 | 0.02, 3.94 | 0.42 | 0.25 | 0.01, 5.08 | | 0.36 | 0.27 | 0.01, 6.97 | 0.43 |  |
| **Animal products by site** | 157 |  |  |  |  |  | |  |  |  |  |  |
| Lower Tercile |  | — | — |  | — | — | |  | — | — |  |  |
| Middle Tercile |  | 0.43 | 0.02, 4.56 | 0.49 | 0.47 | 0.04, 6.16 | | 0.56 | 0.50 | 0.03, 8.78 | 0.63 |  |
| Upper Tercile |  | 0.59 | 0.03, 7.09 | 0.69 | 0.35 | 0.02, 5.62 | | 0.46 | 0.35 | 0.02, 6.37 | 0.48 |  |
| **Roots, tubers & plantain by site** | 157 |  |  |  |  |  | |  |  |  |  |  |
| Lower Tercile |  | — | — |  | — | — | |  | — | — |  |  |
| Middle Tercile |  | 0.00 | 0.00, Inf | 1.00 | 0.00 | 0.00, Inf | | 1.00 | 0.00 | 0.00, Inf | 1.00 |  |
| Upper Tercile |  | 0.31 | 0.03, 2.97 | 0.31 | 0.20 | 0.01, 3.75 | | 0.28 | 0.18 | 0.01, 5.23 | 0.32 |  |
| *^1^* IRR = Incidence Rate Ratio, CI = Confidence Interval — means reference.  Model 1: age, sex, Model 2: Model 1 plus education (never or elementary; low; intermediate; high vocational), total energy intake (kcal per day), smoking (never; former; current), physical activity (METs-h per week). Model 3: Model 2 plus BMI (kg/m2), waist-hip-ratio, Total cholesterol (mmol/L), SBP (mmHg) | | | | | | | | | | | | |

| **Supplementary Table 2F: Association between Baseline dietary patterns and Incidence diabetes by sex** - **Amsterdam (male)** | | | | | | | | | | | | | |
| --- | --- | --- | --- | --- | --- | --- | --- | --- | --- | --- | --- | --- | --- |
| **Dietary Pattern** | **Model 1** | | | | **Model 2** | | | | **Model 3** | | | |  |
|  | **N** | **IRR***^1^* | **95% CI***^1^* | **p-value** | | **IRR***^1^* | **95% CI***^1^* | **p-value** | | **IRR***^1^* | **95% CI***^1^* | **p-value** | |
| **Mixed dietary pattern by site** | 123 |  |  |  | |  |  |  | |  |  |  | |
| Lower Tercile |  | — | — |  | | — | — |  | | — | — |  | |
| Middle Tercile |  | 0.48 | 0.02, 3.78 | 0.53 | | 0.29 | 0.02, 4.64 | 0.38 | | 0.36 | 0.02, 6.76 | 0.50 | |
| Upper Tercile |  | 0.51 | 0.07, 3.12 | 0.47 | | 0.14 | 0.01, 2.15 | 0.16 | | 0.16 | 0.01, 2.78 | 0.21 | |
| **Animal products by site** | 123 |  |  |  | |  |  |  | |  |  |  | |
| Lower Tercile |  | — | — |  | | — | — |  | | — | — |  | |
| Middle Tercile |  | 0.56 | 0.03, 6.04 | 0.64 | | 0.64 | 0.04, 9.28 | 0.75 | | 0.55 | 0.03, 8.88 | 0.68 | |
| Upper Tercile |  | 1.85 | 0.29, 14.6 | 0.51 | | 1.09 | 0.13, 9.13 | 0.94 | | 0.89 | 0.09, 8.85 | 0.92 | |
| **Roots, tubers & plantain by site** | 123 |  |  |  | |  |  |  | |  |  |  | |
| Lower Tercile |  | — | — |  | | — | — |  | | — | — |  | |
| Middle Tercile |  | 3.10 | 0.40, 62.7 | 0.33 | | 3.98 | 0.35, 45.7 | 0.27 | | 4.96 | 0.39, 62.4 | 0.22 | |
| Upper Tercile |  | 2.36 | 0.23, 50.7 | 0.48 | | 1.96 | 0.15, 25.5 | 0.61 | | 2.29 | 0.17, 31.4 | 0.54 | |
| *^1^* IRR = Incidence Rate Ratio, CI = Confidence Interval — means reference.  Model 1: age, sex, Model 2: Model 1 plus education (never or elementary; low; intermediate; high vocational), total energy intake (kcal per day), smoking (never; former; current), physical activity (METs-h per week). Model 3: Model 2 plus BMI (kg/m2), waist-hip-ratio, Total cholesterol (mmol/L), SBP (mmHg) | | | | | | | | | | | | | |

| **Characteristic** | **Model 1** | | | | **Model 2** | | | | **Model 3** | | |  |
| --- | --- | --- | --- | --- | --- | --- | --- | --- | --- | --- | --- | --- |
|  | **N** | **IRR***^1^* | **95% CI***^1^* | **p-value** | | **IRR***^1^* | **95% CI***^1^* | **p-value** | | **IRR***^1^* | **95% CI***^1^* | **p-value** |
| **Mixed dietary pattern** | 463 |  |  |  | |  |  |  | |  |  |  |
| Lower Tercile |  | — | — |  | | — | — |  | | — | — |  |
| Middle Tercile |  | 2.11 | 0.41, 15.2 | 0.39 | | 1.99 | 0.36, 11.0 | 0.43 | | 2.04 | 0.34, 12.3 | 0.43 |
| Upper Tercile |  | 2.97 | 0.72, 20.0 | 0.17 | | 3.79 | 0.73, 19.6 | 0.11 | | 2.79 | 0.52, 15.0 | 0.23 |
| **Animal products** | 463 |  |  |  | |  |  |  | |  |  |  |
| Lower Tercile |  | — | — |  | | — | — |  | | — | — |  |
| Middle Tercile |  | 1.16 | 0.30, 4.75 | 0.83 | | 1.05 | 0.27, 4.02 | 0.94 | | 0.88 | 0.23, 3.43 | 0.85 |
| Upper Tercile |  | 0.93 | 0.21, 4.13 | 0.93 | | 0.69 | 0.16, 3.00 | 0.63 | | 0.48 | 0.10, 2.25 | 0.35 |
| **Roots, tubers & plantain** | 463 |  |  |  | |  |  |  | |  |  |  |
| Lower Tercile |  | — | — |  | | — | — |  | | — | — |  |
| Middle Tercile |  | 0.14 | 0.01, 0.79 | 0.07 | | 0.11 | 0.01, 0.88 | 0.04 | | 0.16 | 0.02, 1.35 | 0.09 |
| Upper Tercile |  | 0.71 | 0.21, 2.22 | 0.55 | | 0.61 | 0.16, 2.28 | 0.44 | | 0.94 | 0.24, 3.73 | 0.93 |
| *^1^* IRR = Incidence Rate Ratio, CI = Confidence Interval — means reference.  Model 1: age, sex, Model 2: Model 1 plus education (never or elementary; low; intermediate; high vocational), total energy intake (kcal per day), smoking (never; former; current), physical activity (METs-h per week). Model 3: Model 2 plus BMI (kg/m2), waist-hip-ratio, Total cholesterol (mmol/L), SBP (mmHg) | | | | | | | | | | | | |
|  |  |  |  |  | |  |  |  | |  |  |  |
|  |  |  |  |  | |  |  |  | |  |  |  |

**Supplementary Table 2G: Association between Baseline dietary patterns and Incidence diabetes by sex - All Sites (Male)**

| **Supplementary Table 3 Interaction effect of sex and animal product dietary patterns with incidence of T2DM** | | | |
| --- | --- | --- | --- |
| **Characteristic** | **IRR***^1^* | **95% CI***^1^* | **p-value** |
|  |  |  |  |
| Sex |  |  |  |
| Male | — | — |  |
| Female | 0.70 | 0.23, 2.31 | 0.53 |
| Animal products by site |  |  |  |
| Lower Tercile | — | — |  |
| Middle Tercile | 0.50 | 0.10, 2.06 | 0.35 |
| Upper Tercile | 0.84 | 0.23, 3.02 | 0.78 |
| Sex * Animal products by site |  |  |  |
| Female * Middle Tercile | 6.76 | 1.36, 39.5 | **0.02** |
| Female * Upper Tercile | 2.11 | 0.45, 9.99 | 0.33 |
| *^1^* IRR = Incidence Rate Ratio, CI = Confidence Interval — means reference.  Model 1: age, sex, Model 2: Model 1 plus education (never or elementary; low; intermediate; high vocational), total energy intake (kcal per day), smoking (never; former; current), physical activity (METs-h per week). Model 3: Model 2 plus BMI (kg/m2), waist-hip-ratio, Total cholesterol (mmol/L), SBP (mmHg) | | | |
|  | | | |

**Supplementary Table 4A: Association between Baseline dietary patterns and Incidence diabetes by sex- All Sites (Female)**

| **Characteristic** | **Model 1** | | | | **Model 2** | | | | **Model 3** | | |  |
| --- | --- | --- | --- | --- | --- | --- | --- | --- | --- | --- | --- | --- |
|  | **N** | **IRR***^1^* | **95% CI***^1^* | **p-value** | | **IRR***^1^* | **95% CI***^1^* | **p-value** | | **IRR***^1^* | **95% CI***^1^* | **p-value** |
| **Mixed dietary pattern** | 890 |  |  |  | |  |  |  | |  |  |  |
| Lower Tercile |  | — | — |  | | — | — |  | | — | — |  |
| Middle Tercile |  | 1.53 | 0.74, 3.26 | 0.26 | | 1.50 | 0.72, 3.15 | 0.28 | | 1.57 | 0.74, 3.32 | 0.24 |
| Upper Tercile |  | 1.46 | 0.69, 3.17 | 0.32 | | 1.63 | 0.75, 3.56 | 0.22 | | 1.68 | 0.76, 3.72 | 0.20 |
| **Animal products** | 890 |  |  |  | |  |  |  | |  |  |  |
| Lower Tercile |  | — | — |  | | — | — |  | | — | — |  |
| Middle Tercile |  | 5.09 | 2.09, 15.2 | <0.001 | | 5.30 | 2.00, 14.0 | <0.001 | | 5.36 | 2.01, 14.3 | **<0.001** |
| Upper Tercile |  | 4.33 | 1.69, 13.3 | 0.01 | | 4.86 | 1.72, 13.7 | <0.001 | | 5.01 | 1.73, 14.6 | **<0.001** |
| **Roots, tubers & plantain** | 890 |  |  |  | |  |  |  | |  |  |  |
| Lower Tercile |  | — | — |  | | — | — |  | | — | — |  |
| Middle Tercile |  | 1.63 | 0.83, 3.32 | 0.17 | | 1.51 | 0.75, 3.02 | 0.25 | | 1.49 | 0.74, 3.00 | 0.27 |
| Upper Tercile |  | 0.85 | 0.37, 1.89 | 0.68 | | 0.75 | 0.29, 1.92 | 0.54 | | 0.83 | 0.31, 2.17 | 0.70 |
| *^1^* IRR = Incidence Rate Ratio, CI = Confidence Interval — means reference.  Model 1: age, sex, Model 2: Model 1 plus education (never or elementary; low; intermediate; high vocational), total energy intake (kcal per day), smoking (never; former; current), physical activity (METs-h per week). Model 3: Model 2 plus BMI (kg/m2), waist-hip-ratio, Total cholesterol (mmol/L), SBP (mmHg) | | | | | | | | | | | | |
|  |  |  |  |  | |  |  |  | |  |  |  |

| **Supplementary Table 4B.**  **Interaction effect of sex and mixed dietary patterns with incidence of T2DM** | | | |
| --- | --- | --- | --- |
| **Characteristic** | **IRR***^1^* | **95% CI***^1^* | **p-value** |
| Sex |  |  |  |
| Male | — | — |  |
| Female | 1.56 | 0.61, 4.78 | 0.38 |
| Mixed dietary pattern by site |  |  |  |
| Lower Tercile | — | — |  |
| Middle Tercile | 0.96 | 0.27, 3.46 | 0.95 |
| Upper Tercile | 0.55 | 0.11, 2.25 | 0.41 |
| Sex * Mixed dietary pattern by site |  |  |  |
| Female * Middle Tercile | 0.86 | 0.20, 3.73 | 0.84 |
| Female * Upper Tercile | 2.01 | 0.42, 11.1 | 0.39 |
| *^1^* IRR = Incidence Rate Ratio, CI = Confidence Interval — means reference.  Model 1: age, sex, Model 2: Model 1 plus education (never or elementary; low; intermediate; high vocational), total energy intake (kcal per day), smoking (never; former; current), physical activity (METs-h per week). Model 3: Model 2 plus BMI (kg/m2), waist-hip-ratio, Total cholesterol (mmol/L), SBP (mmHg) | | | |

| **Supplementary Table 4C Interaction effect of sex and roots, tubers and plantain dietary patterns with incidence of T2DM** | | | |
| --- | --- | --- | --- |
| **Characteristic** | **IRR***^1^* | **95% CI***^1^* | **p-value** |
|  |  |  |  |
| Sex |  |  |  |
| Male | — | — |  |
| Female | 1.24 | 0.46, 3.91 | 0.68 |
| Roots, tubers & plantain by site |  |  |  |
| Lower Tercile | — | — |  |
| Middle Tercile | 0.56 | 0.12, 2.30 | 0.43 |
| Upper Tercile | 1.06 | 0.30, 3.82 | 0.92 |
| Sex * Roots, tubers & plantain by site |  |  |  |
| Female * Middle Tercile | 2.76 | 0.57, 15.6 | 0.21 |
| Female * Upper Tercile | 1.22 | 0.28, 5.34 | 0.79 |
| *^1^* IRR = Incidence Rate Ratio, CI = Confidence Interval — means reference.  Model 1: age, sex, Model 2: Model 1 plus education (never or elementary; low; intermediate; high vocational), total energy intake (kcal per day), smoking (never; former; current), physical activity (METs-h per week). Model 3: Model 2 plus BMI (kg/m2), waist-hip-ratio, Total cholesterol (mmol/L), SBP (mmHg) | | | |
|  | | | |

**Supplementary Table 5A Interaction effect of site and mixed dietary patterns with incidence of T2DM**

| **Characteristic** | **IRR***^1^* | **95% CI***^1^* | **p-value** |
| --- | --- | --- | --- |
| Site |  |  |  |
| Rural Ghana | — | — |  |
| Urban Ghana | 2.42 | 0.81, 8.81 | 0.14 |
| Amsterdam | 2.91 | 0.88, 11.1 | 0.09 |
| Mixed dietary pattern by site |  |  |  |
| Lower Tercile | — | — |  |
| Middle Tercile | 1.00 | 0.24, 4.23 | >1.00 |
| Upper Tercile | 1.00 | 0.24, 4.23 | >1.00 |
| Site * Mixed dietary pattern by site |  |  |  |
| Urban Ghana * Middle Tercile | 1.00 | 0.19, 5.35 | >1.00 |
| Amsterdam * Middle Tercile | 0.57 | 0.08, 3.67 | 0.55 |
| Urban Ghana * Upper Tercile | 1.20 | 0.23, 6.31 | 0.83 |
| Amsterdam * Upper Tercile | 0.57 | 0.08, 3.67 | 0.55 |
| *^1^* *^1^* IRR = Incidence Rate Ratio, CI = Confidence Interval — means reference.  Model 1: age, sex, Model 2: Model 1 plus education (never or elementary; low; intermediate; high vocational), total energy intake (kcal per day), smoking (never; former; current), physical activity (METs-h per week). Model 3: Model 2 plus BMI (kg/m2), waist-hip-ratio, Total cholesterol (mmol/L), SBP (mmHg) | | | |

**Supplementary Table 5B Interaction effect of site and animal products dietary patterns with incidence of T2DM**

| **Characteristic** | **IRR***^1^* | **95% CI***^1^* | **p-value** |
| --- | --- | --- | --- |
| Site |  |  |  |
| Rural Ghana | — | — |  |
| Urban Ghana | 2.25 | 0.63, 10.5 | 0.24 |
| Amsterdam | 1.66 | 0.31, 8.97 | 0.53 |
| Animal products by site |  |  |  |
| Lower Tercile | — | — |  |
| Middle Tercile | 2.00 | 0.53, 9.48 | 0.33 |
| Upper Tercile | 1.00 | 0.19, 5.40 | >1.00 |
| Site * Animal products by site |  |  |  |
| Urban Ghana * Middle Tercile | 1.07 | 0.18, 5.45 | 0.93 |
| Amsterdam * Middle Tercile | 1.17 | 0.16, 8.59 | 0.88 |
| Urban Ghana * Upper Tercile | 1.43 | 0.21, 9.89 | 0.71 |
| Amsterdam * Upper Tercile | 1.67 | 0.19, 15.7 | 0.64 |
| *^1^* IRR = Incidence Rate Ratio, CI = Confidence Interval — means reference.  Model 1: age, sex, Model 2: Model 1 plus education (never or elementary; low; intermediate; high vocational), total energy intake (kcal per day), smoking (never; former; current), physical activity (METs-h per week). Model 3: Model 2 plus BMI (kg/m2), waist-hip-ratio, Total cholesterol (mmol/L), SBP (mmHg) | | | |

**Supplementary Table 5C Interaction effect of site and roots, tubers and plantain products dietary patterns with incidence of T2DM**

| **Characteristic** | **IRR***^1^* | **95% CI***^1^* | **p-value** |
| --- | --- | --- | --- |
| Site |  |  |  |
| Rural Ghana | — | — |  |
| Urban Ghana | 1.74 | 0.60, 5.66 | 0.32 |
| Amsterdam | 1.00 | 0.20, 4.06 | 1.00 |
| Roots, tubers & plantain by site |  |  |  |
| Lower Tercile | — | — |  |
| Middle Tercile | 0.60 | 0.12, 2.45 | 0.48 |
| Upper Tercile | 0.80 | 0.20, 3.02 | 0.74 |
| Site * Roots, tubers & plantain by site |  |  |  |
| Urban Ghana * Middle Tercile | 2.22 | 0.43, 13.3 | 0.35 |
| Amsterdam * Middle Tercile | 3.33 | 0.48, 28.1 | 0.24 |
| Urban Ghana * Upper Tercile | 1.53 | 0.31, 7.88 | 0.60 |
| Amsterdam * Upper Tercile | 2.50 | 0.38, 18.9 | 0.35 |
| *^1^* *^1^* IRR = Incidence Rate Ratio, CI = Confidence Interval — means reference.  Model 1: age, sex, Model 2: Model 1 plus education (never or elementary; low; intermediate; high vocational), total energy intake (kcal per day), smoking (never; former; current), physical activity (METs-h per week). Model 3: Model 2 plus BMI (kg/m2), waist-hip-ratio, Total cholesterol (mmol/L), SBP (mmHg) | | | |
